# Case Report: *MYBPC1*-associated congenital myopathy with tremor (CMYO16): a multigenerational family report

**DOI:** 10.3389/fgene.2026.1922946

**Published:** 2026-10-30

**Authors:** Leping Li, Jenna K. Lea, Hadley J. Bryan, Nathaniel G. Wooten, Steven P. Trau, Xiaoling Li, Zheng Fan

**Affiliations:** 1 Biostatistics and Computational Biology Branch, National Institute of Environmental Health Sciences, Durham, NC, United States; 2 Department of Neurology, University of North Carolina at Chapel Hill, Chapel Hill, NC, United States; 3 Division of Pediatric Neurology, Department of Neurology, University of North Carolina at Chapel Hill, Chapel Hill, NC, United States; 4 Molecular and Cellular Biology Laboratory, National Institute of Environmental Health Sciences, Durham, NC, United States

**Keywords:** acetylcholinesterase inhibitor, CHRND, CMYO16, congenital myopathy, MYBPC1, myogenic tremor, pyridostigmine

## Abstract

We report a family with congenital myopathy-16 (CMYO16; MIM 618524) due to a novel heterozygous *MYBPC1* M-motif variant, c.779G>A p (Arg260His), in whom oral pyridostigmine was associated with apparent clinical benefit in all four affected members across three generations. To our knowledge, this is the first report of a possible clinical response to pyridostigmine in *MYBPC1*-associated congenital myopathy with tremor. The *MYBPC1* gene encodes slow skeletal myosin-binding protein C (sMyBP-C), a sarcomeric accessory protein that regulates actomyosin cross-bridge cycling and stabilizes the thick filament. Heterozygous variants, most commonly involving the highly conserved M-motif, cause CMYO16, a mild, early-onset, slowly progressive disorder characterized by hypotonia, fatigability, proximal and axial weakness, and a distinctive high-frequency tremor. Few than thirty affected individuals have been reported, and no disease-specific or symptom-directed pharmacologic therapy has been described. In this family, the variant co-segregated with the phenotype in four affected individuals: a young boy (the proband), his father, paternal aunt, and paternal grandfather. Because of disabling fatigability, clinical features suggestive of congenital myasthenia, and the presence of a *CHRND* variant of uncertain significance in three family members, a trial of oral pyridostigmine was initiated in the proband at 16 months of age. A blinded one-week-on/one-week-off assessment by a physical therapist suggested improvement in tone, energy, and gait, and caregivers reported improved stamina and reduced tremors. Objective assessments could not be obtained because of the patient’s young age. Concurrent improvement in gastrointestinal symptoms most likely reflects the known prokinetic cholinergic effects of pyridostigmine rather than a muscle-specific benefit. Open-label, self-reported benefit was subsequently described by the three affected adults. Because the outcomes were open-label and largely subjective, these observations are preliminary and hypothesis-generating. The affected paternal aunt did not carry the *CHRND* variant, arguing against attributing the response to *CHRND* alone; however, her prior diagnosis of fibromyalgia makes her self-reported improvement particularly susceptible to placebo effects. We report that four affected family members, ranging in age from 2 to 65 years, showed a positive and well-tolerated response to pyridostigmine, with improvement in clinical symptoms likely attributable to CMYO16.

## Introduction

The myosin-binding protein C (MyBP-C) family comprises three accessory sarcomeric proteins—slow skeletal (sMyBP-C), fast skeletal (fMyBP-C), and cardiac (cMyBP-C), encoded by *MYBPC1*, *MYBPC2*, and *MYBPC3*, respectively (Ackermann and Kontrogianni-Konstantopoulos, 2013; Lin et al., 2013). sMyBP-C is a modular protein of seven immunoglobulin and three fibronectin type III domains (C1–C10), interspersed with a proline/alanine-rich region and a MyBP-C–specific segment, the M-motif, which flanks the C1 domain. The M-motif is an evolutionarily conserved and represents a hotspot for disease-associated *MYBPC1* variants (Shashi et al., 2019) (Figure 1). The N-terminus of sMyBP-C binds the myosin S2 subfragment and actin to modulate cross-bridge formation, while the C-terminus anchors the protein to the thick filament.

**FIGURE 1:**
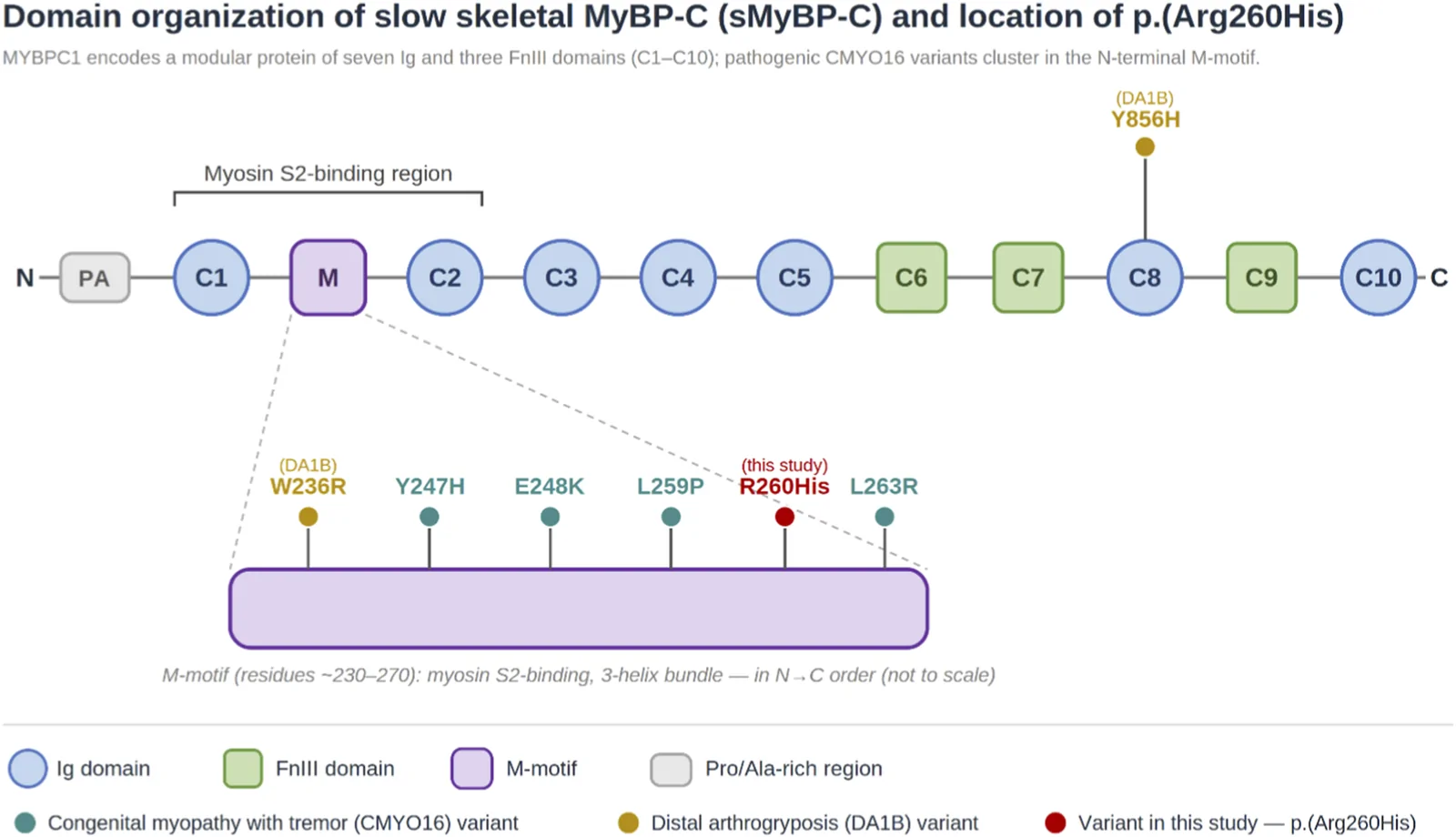
Schematic of the sMyBP-C domain structure encoded by *MYBPC1*, showing the proline/alanine-rich region, the M-motif, and immunoglobulin/fibronectin type III domains C1–C10. The novel p. (Arg260His) variant maps within the M-motif, with the myosin S2-binding region indicated. An expanded view of the M-motif shows p. (Arg260His) (this study, highlighted) among the previously reported myopathy-with-tremor variants W236R, Y247H, E248K, L259R, and L263R in N-to-C order.

Biallelic loss-of-function defects in *MYBPC1* cause lethal congenital contracture syndrome 4 and arthrogryposis multiplex congenita (Markus et al., 2012; Ekhilevitch et al., 2016), whereas heterozygous damaging variants produce distal arthrogryposis type 1B or an early-onset congenital myopathy with tremor (CMYO16; MIM 618524) (Shashi et al., 2019; Bhandari et al., 2024; Uneoka et al., 2023; Shiraishi et al., 2022; Leduc-Pessah et al., 2024; Li et al., 2015; Geist Hauserman et al., 2021; Stavusis et al., 2019; Gurnett et al., 2010). *MYBPC1* was first linked to autosomal dominant distal arthrogryposis in 2010 (Gurnett et al., 2010), and the expanded, contracture-free myopathic-with-tremor phenotype was delineated in 2019 (Shashi et al., 2019; Stavusis et al., 2019). CMYO16 is a mild and slowly progressive myopathy characterized by neonatal hypotonia, delayed motor milestones, fatigability, mild axial and proximal weakness, and a distinctive high-frequency postural and appendicular tremor that frequently involves the tongue. This tremor is widely regarded as myogenic in origin (Shashi et al., 2019; Geist Hauserman et al., 2021; Stavusis et al., 2019; Mariano et al., 2025). Most reported pathogenic variants cluster within the N-terminal M-motif, where they reduce binding of sMyBP-C to myosin thereby impairing cross-bridge regulation (Shashi et al., 2019; Geist Hauserman et al., 2021; Stavusis et al., 2019).

To date, fewer than thirty individuals with CMYO16 from only a small number of families have been reported (Bhandari et al., 2024; Uneoka et al., 2023; Shiraishi et al., 2022; Leduc-Pessah et al., 2024; Geist Hauserman et al., 2021; Stavusis et al., 2019; Velardo et al., 2026; Lanvin et al., 2024). Management remains entirely supportive, consisting primarily of physical therapy and, when tremor is disabling, symptomatic measures of uncertain benefit. No treatment specifically targeting the weakness or tremor of CMYO16 has yet been described. Here, we report a three-generation family with a co-segregating heterozygous *MYBPC1* M-motif variant in which all four affected members, including three adults, reported apparent, open-label clinical benefit from pyridostigmine (described in detail, with its limitations, below).

Pyridostigmine (commonly known by the brand name Mestinon) is a reversible acetylcholinesterase inhibitor (Singh and Patel, 2026; Remijn-Nelissen et al., 2026; Remijn-Nelissen et al., 2022). It enhances communication between motor nerves and skeletal muscle by slowing the breakdown of acetylcholine and increasing its availability at the neuromuscular junction. It is commonly used to treat disorders of neuromuscular transmission, particularly myasthenia gravis and certain congenital myasthenic syndromes. If this observation is confirmed in additional patients, pyridostigmine may represent a meaningful therapeutic option for patients with CMYO16. We propose a mechanistic framework for this response and suggest that CMYO16 be added to the growing list of congenital myopathies that may benefit from enhancement of neuromuscular transmission.

## Case description

### Proband

The proband is a boy who first presented to pediatric neurology at 15 months of age with congenital generalized hypotonia, delays in gross and fine motor milestones, and easy fatigability. He was born at 38 weeks’ gestation by emergency cesarean section following a pregnancy complicated by low maternal free thyroxine (levothyroxine started at ∼16 weeks) and maternal sepsis. He required a brief neonatal-unit admission for sepsis surveillance. The newborn screen was normal, and head circumference at birth was 34 cm (36th percentile). Transient neonatal chin twitching prompted an electroencephalogram, which was normal.

In infancy, he had laryngomalacia with noisy breathing attributed to laryngomalacia, prominent feeding difficulties with a hypersensitive gag, cyclic vomiting, and food intolerance (a pediatric feeding disorder), together with constipation; upper gastrointestinal study with biopsies and a modified swallow evaluation were unremarkable. Caregivers and his primary clinician noted reduced spontaneous use of the arms and limited overhead reach from approximately 6 months. He received intensive physical and feeding therapy. Creatine kinase was mildly elevated (approximately 201 U/L). He had an expressive language delay, while receptive language and cognition were preserved.

On examination at approximately 16 months of age, he was alert, visually interactive, cognitively age-appropriate, and speaking in phrases. Mild facial hypotonia, no ptosis. He had mild generalized hypotonia, more pronounced in the trunk than in the appendicular limbs, with normal muscle bulk and largely preserved strength on confrontation testing; fine motor skills were intact. Mild weakness of the shoulder and pelvic girdle muscles was present. Deep tendon reflexes were 1 + throughout, and plantar responses were downgoing. He walked independently with a mildly wide-based, lordotic gait, intermittent toe-walking, and mild in-toeing, greater on the right than the left, and he fell frequently. Relatively mild prominent calf musculature and a predominantly proximal pattern of lower-extremity involvement were noted. No contractures were present.

### Family history

The parents were non-consanguineous. A strong autosomal dominant family history was evident across three generations on the paternal side. The proband’s father (approximately 35 years old), paternal aunt (approximately 30 years old), and paternal grandfather (approximately 65 years old) all reported chronic fatigability, low energy, mild proximal weakness, and a long-standing tremor that had previously been clinically classified as essential tremor. The paternal aunt additionally had reduced muscle mass and carried a diagnosis of fibromyalgia with chronic pain. In contrast, a paternal uncle in his 30s was unaffected and was an accomplished competitive weightlifter. There was no relevant history on the maternal side. These findings are consistent with the mild myopathy-with-tremor phenotype of CMYO16 segregating through the paternal lineage. The pedigree is shown in Figure 2.

**FIGURE 2:**
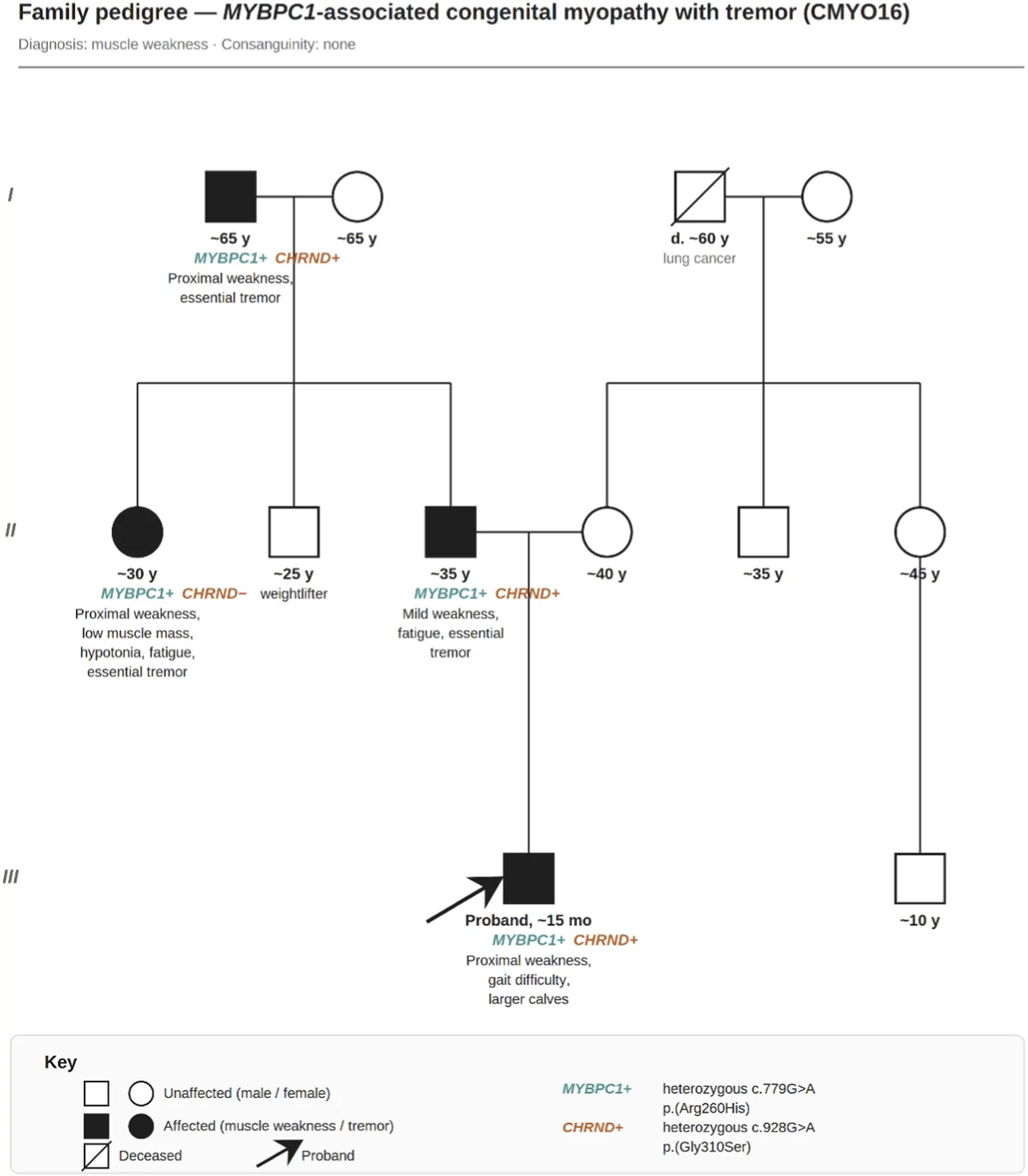
Pedigree of the family. Filled symbols denote clinically affected individuals; the arrow indicates the proband. The heterozygous *MYBPC1* variant c.779G > A p. (Arg260His) is present in all affected members (proband, father, paternal aunt, paternal grandfather), consistent with autosomal dominant inheritance. The *CHRND* variant of uncertain significance p. (Gly310Ser) is present in the proband, father, and paternal grandfather but absent in the affected paternal aunt.

### Genetic testing

A neuromuscular gene panel performed earlier in the proband’s evaluation was non-diagnostic and did not include *MYBPC1*. Subsequent diagnostic exome sequencing with single-gene analysis at GeneDx in the affected paternal aunt identified a heterozygous *MYBPC1* variant, c.779G > A p. (Arg260His), in exon 11 (NM_002465.3), which was classified as likely pathogenic for *MYBPC1*-related congenital myopathy. For *MYBPC1*, the sequencing data showed no evidence of a multi-exon deletion or duplication involving this gene. Targeted testing confirmed the same variant in the proband and his father. The variant co-segregated with the phenotype in all four available affected family members: the proband, his father, paternal aunt, and paternal grandfather. Genetic testing was not performed in the paternal uncle, the grandmother, or any maternal relatives.

To our knowledge, the *MYBPC1* M-motif variant, c.779G > A p. (Arg260His) has not been previously reported. It is located within the N-terminal M-motif, immediately adjacent to the previously characterized pathogenic residues p. Leu259Pro and p. Leu263Arg, in the third helix of the M-motif three-helix bundle (Figure 1). Protein modeling studies have suggested that the p. (Leu263Arg) substitution disrupts M-motif stability, whereas the p. (Leu259Pro) substitution likely alters the structural integrity of the motif (Shashi et al., 2019).

A heterozygous variant of uncertain significance in *CHRND*, c.928G > A p. (Gly310Ser) (rs771670415), was also identified in the proband, his father, and the paternal grandfather, and was absent in the affected paternal aunt. This variant is rare in gnomAD (minor allele frequency on the order of 1–7 × 10^-5^) and is catalogued in ClinVar as a variant of uncertain significance (VCV001020812). *CHRND* encodes the acetylcholine receptor δ subunit, and pathogenic *CHRND* variants can cause congenital myasthenic syndromes. However, in the absence of functional data, p. (Gly310Ser) meets criteria only for a variant of uncertain significance, and no pathogenic or modifier effect can be assigned to it. Its distribution within the family is discussed below in relation to the treatment response; critically, it does not co-segregate with affected status, whereas the *MYBPC1* variant does.

### Therapeutic intervention and response

Given the disabling fatigability and the absence of any established disease-directed therapy, a trial of oral pyridostigmine was initiated in the proband. This decision was supported by clinical features suggestive of a congenital myasthenic syndrome and by the presence of a *CHRND* variant of uncertain significance. Pyridostigmine was started at 16 months of age at 7 mg/kg/day in three divided doses and was subsequently increased to 8 mg/kg/day in three divided doses. As part of our standard approach to empirical medication trials, treatment response was assessed in a blinded fashion by a physical therapist. During the initial trial, a pediatric physical therapist, blinded to dosing status, performed a 2-week qualitative assessment consisting of 1 week on medication and 1 week off medication. This evaluation showed improvement in tone, energy, activity level, and gait stability while the patient was receiving pyridostigmine. Because of the patient’s young age, quantitative measures such as formal strength testing were not performed. The therapist also noted that he was able to complete therapy sessions that he had previously been unable to finish. At the time of this report, the proband had been receiving pyridostigmine for approximately 2 years, with continued reported benefit.

Caregivers reported clear and rapid improvement in muscle tone, energy, and physical stamina, with increased overhead use of the arms and fewer falls. They also reported improvement in gastrointestinal symptoms, including reduced constipation and less frequent episodes of cyclic vomiting; this is most plausibly explained by the known prokinetic action of pyridostigmine, a generalized cholinergic effect on gut smooth muscle, rather than by a disease-specific effect on the myopathy, and it should not be interpreted as evidence of efficacy against CMYO16. The effect wore off predictably shortly before each scheduled dose, with re-emergence of fatigue, and symptoms improved again after dosing. No adverse effects were observed.

Because of the dramatic response to pyridostigmine in the proband, treatment trials were also initiated in the three affected adults. All three reported similar benefit. Needle EMG performed in the father 2 years earlier showed mild myopathic motor unit changes without abnormal spontaneous activity in the right deltoid and vastus lateralis, consistent with mild chronic myopathy. In the father, repetitive nerve stimulation at 2 Hz of the right ulnar and facial nerves showed no decremental response at baseline or after exercise. Clinically, he reported clear improvement in distal tremor and endurance with pyridostigmine compared with baseline. The paternal aunt, whose chronic pain had previously been attributed to fibromyalgia, reported a marked reduction in tremor, substantial improvement in energy, and decreased chronic pain, enabling her to begin exercising regularly; she described the treatment as having “given her life back”. The paternal aunt is currently taking high-dose pyridostigmine, 120 mg five times daily (approximately 600 mg/day). The paternal grandfather was also reported to have responded well, although detailed clinical records were not available. The paternal grandfather and paternal aunt live overseas; therefore, we were unable to examine them. A trial of albuterol was undertaken because β2-agonists are commonly used in selected congenital myasthenic syndromes. A trial of L-carnitine was also initiated because the proband’s laboratory testing showed low to borderline total carnitine levels in the 30s (μmol/L). Both trials in the proband and father did not produce clear clinical benefit. The clinical features and treatment responses across the family are summarized in Table 1.

**TABLE 1:** Clinical features and response to pyridostigmine across the four affected family members.

Feature	Proband	Father	Paternal aunt	Paternal grandfather
Age	16 months at presentation	∼40 years	∼30 years	∼65 years
*MYBPC1* p. (Arg260His)	Present	Present	Present	Present
*CHRND* p. (Gly310Ser)	Present	Present	Absent	Present
Hypotonia	Mild–moderate	Mild	Mild	Mild
Weakness	Girdle weakness	Low endurance	Low energy	Low energy
Tremor	Neonatal chin twitching	Distal tremor	Prominent tremor	Tremor
Other features	Feeding/GI issues, stridor, mild ↑CK	Minimum	Chronic pain (“fibromyalgia”)	Minimum
Pyridostigmine response	Marked: tone, stamina, GI; PT-confirmed	Good: tremor, endurance	Marked: tremor, energy, pain	Reported good response
Pyridostigmine dose	8 mg/kg/d div tid	120 mg taken 1–2 h before exercise	120 mg five times a day	N/D

CK, creatine kinase; GI, gastrointestinal; PT, physical therapy; Adult relatives were assessed clinically and by self-report; the proband was additionally evaluated by physical therapy.

## Discussion

We describe a multigenerational family with a co-segregating heterozygous *MYBPC1* M-motif variant, c.779G > A p. (Arg260His), and a clinical phenotype typical of CMYO16 (Shashi et al., 2019; Ekhilevitch et al., 2016; Stavusis et al., 2019; Velardo et al., 2026). The M-motif is an evolutionarily conserved region and a recognized hotspot for disease-associated *MYBPC1* variants (Shashi et al., 2019) (Figure 1). Several substitutions in close proximity to this residue, including p. (Trp236Arg), p. (Tyr247His), p. (Glu248Lys), p. (Leu259Pro), and p. (Leu263Arg), have been shown to disrupt muscle function and are associated with congenital myopathy with tremor or distal arthrogryposis. Protein modeling studies have suggested that p. (Leu263Arg) disrupts M-motif stability, whereas p. (Leu259Pro) likely impairs the structural integrity of the motif (Shashi et al., 2019). More recently, biophysical and structural analyses of a non-missense variant, c.795_803dup p. (Leu266_Lys267_Arg268dup), suggested that the duplicated residues directly contribute to Myotrem by increasing M-domain conformational dynamics, enhancing myosin binding, and altering interactions with neighboring and more distant residues (Iyer et al., 2026). Although p. (Arg260His) has not, to our knowledge, been previously reported, its location within this mutational hotspot, together with the known pathogenicity of neighboring residues, strongly supports its clinical significance. Structurally, Arg260 lies within the third helix of the M-motif three-helix bundle and is predicted to participate in a helix-stabilizing hydrogen-bond network; substitution with histidine would therefore be expected to perturb local helix stability in a manner analogous to nearby pathogenic M-motif variants.

Based on these evidence, applying the ACMG/AMP criteria (Richards et al., 2015), we classify c.779G > A p. (Arg260His) as likely pathogenic based on co-segregation with the phenotype in four affected individuals across three generations, with absence in tested unaffected relatives (PP1); absence from population databases, including gnomAD v4.1.2 (PM2_supporting); localization within the established M-motif mutational hotspot immediately adjacent to previously reported pathogenic residues (PM1) (Shashi et al., 2019; Geist Hauserman et al., 2021; Stavusis et al., 2019); and concordant *in silico* predictions supporting a damaging effect (PP3) (Iyer et al., 2026). Because c.779G > A affects a CpG dinucleotide, a site prone to recurrent G > A transitions, its population frequency should be confirmed rather than presumed to indicate novelty. Functional characterization has not been performed, and the variant classification was reviewed with clinical genetics.

The principal and novel observation is that a trial of pyridostigmine, a reversible acetylcholinesterase inhibitor, was associated with apparent clinical benefit in all affected family members - the young proband and the three affected adults. The reported improvement was open-label and included weakness, fatigability, tremor, and related functional symptoms. To our knowledge, no pharmacologic therapy has previously been reported to improve the weakness, fatigability, or tremor associated with CMYO16. Although preliminary, this observation is clinically relevant because it identifies a readily available, inexpensive, and familiar agent as a potential symptomatic treatment for a condition that is currently managed with supportive care alone.

### A plausible link to the tremor

The improvement in tremor is notable, as the CMYO16 tremor is thought to be myogenic, arising from spontaneous pulsatile sarcomere oscillations and exaggerated stretch activation in muscle fibers expressing mutant sMyBP-C, and propagated through a sensorimotor feedback loop (Ekhilevitch et al., 2016; Geist Hauserman et al., 2021; Mariano et al., 2025). It is unproven whether or how pyridostigmine would influence such a tremor; one speculative possibility is that enhanced cholinergic drive could alter calcium-activated cross-bridge occupancy and thereby modulate these oscillations. This remains a hypothesis without direct supporting evidence and is offered only to guide future study; it is consistent with the previously proposed sarcomeric model of myogenic (“myotremor”) tremor (Geist Hauserman et al., 2021; Mariano et al., 2025; Schaefer et al., 2021).

### Interpreting the co-existing *CHRND* variant

The family also segregates a *CHRND* variant of uncertain significance, p. (Gly310Ser). Because *CHRND* encodes the acetylcholine receptor δ subunit and pathogenic *CHRND* variants can cause pyridostigmine-responsive congenital myasthenic syndromes (Engel et al., 2015), it is important to consider whether the observed drug response might instead reflect an unrecognized myasthenic disorder. However, two observations argue against attributing the response to *CHRND* alone. First, the *CHRND* variant does not co-segregate with the phenotype: the affected paternal aunt does not carry it, yet she showed one of the most striking responses to pyridostigmine, whereas the *MYBPC1* variant is present in all affected family members. Second, the proband’s clinical picture, congenital hypotonia, mild myopathy, elevated creatine kinase, and tremor, is more consistent with CMYO16 than with a classic congenital myasthenic syndrome. Taken together, these findings indicate that the pyridostigmine response tracks with the *MYBPC1*-associated myopathy. That the affected paternal aunt does not carry the *CHRND* variant yet reported benefit argues against attributing the response to *CHRND*. The central point, however, is that p. (Gly310Ser) is a single heterozygous variant of uncertain significance without functional data, and no pathogenic or modifier role can currently be assigned to it. Its interpretation was reviewed with clinical genetics, and identification of the variant in a clinically unaffected relative would further support its non-pathogenicity. As noted in the Limitations, the aunt’s response is also the most susceptible to placebo effects.

### Why might an acetylcholinesterase inhibitor help a sarcomeric myopathy?

A response to pyridostigmine may seem unexpected in a sarcomeric disorder, as sMyBP-C has no established role at the neuromuscular junction (NMJ) and is primarily recognized as a skeletal-muscle sarcomeric protein rather than a neuronal or peripheral nerve protein (Ekhilevitch et al., 2016; Stavusis et al., 2019). A response to pyridostigmine does not, however, establish that the tremor is myogenic in origin. Indeed, several congenital myopathies are now known to have secondary defects of neuromuscular transmission that can be improved with cholinesterase inhibition, including TPM2-related cap myopathy, DNM2-and MTM1-related centronuclear/myotubular myopathies, and RYR1-related myopathy (Singh and Patel, 2026; Schaefer et al., 2021; Robb et al., 2011; Rodriguez Cruz et al., 2014; Gibbs et al., 2013). In these disorders, endplate abnormalities reduce the safety margin of transmission and contribute a fatigable, treatment-responsive component to weakness (Engel et al., 2015; Robb et al., 2011; Rodriguez Cruz et al., 2014). Pyridostigmine increases acetylcholine availability at the NMJ, enhancing and prolonging endplate depolarization (Singh and Patel, 2026; Remijn-Nelissen et al., 2026; Remijn-Nelissen et al., 2022). In CMYO16, where M-motif variants impair myosin binding and cross-bridge function (Shashi et al., 2019; Iyer et al., 2026), pyridostigmine-induced increase of cholinergic drive could partly compensate this deficit by recruiting more cross-bridges per impulse and sustaining activation, thereby improving strength, stamina, and fatigability. Whether *MYBPC1* myopathy could also lead to secondary endplate remodeling, as in other congenital myopathies, remains to be determined.

We emphasize that any role for sMyBP-C near the subsynaptic apparatus is purely speculative and was not tested in this study. However, current evidence localizes sMyBP-C to the sarcomere rather than the NMJ (Ekhilevitch et al., 2016; Stavusis et al., 2019), making a secondary transmission defect the more parsimonious explanation unless future immunolocalization and electrophysiologic studies suggest otherwise. The possibility that pyridostigmine alters the interaction between mutant sMyBP-C and myosin is likewise speculative and, based on current evidence, unlikely.

### Diagnostic and management implications

Our observations have several practical implications. From a diagnostic standpoint, the *MYBPC1* variant was not included on the initial targeted neuromuscular panel and identified only through exome sequencing performed in an affected relative, highlighting the value of broad, hypothesis-free genomic testing in congenital hypotonia and the importance of family-based evaluation. Given the father’s normal repetitive nerve stimulation study, a defect in neuromuscular transmission is less strongly supported as the basis for pyridostigmine benefit. A carefully monitored, individualized trial of pyridostigmine may be considered in symptomatic CMYO16, with attention to dose-related wearing off and standard cholinergic precautions. The lack of clear benefit from albuterol in our proband does not exclude impaired neuromuscular transmission, as responses to β2-agonist therapy are variable across these disorders (Rodriguez Cruz et al., 2014). Finally, given the reported respiratory and cardiac manifestations within the broader *MYBPC1* disease spectrum, periodic respiratory and cardiac surveillance remains advisable (Velardo et al., 2026).

### Limitations

This report has several important limitations. First, the observations were open-label and relied largely on clinician-, caregiver-, and patient-reported outcomes without a uniform objective endpoint; therefore, placebo or expectation effects cannot be excluded. Strength and function were not assessed using standardized measures applied consistently across all four affected individuals. Descriptive terms such as “low energy,” “low endurance,” and “low muscle mass” do not constitute formal strength assessments, and the reported improvements are therefore not readily reproducible or comparable across individuals. The proband underwent only a brief 2-week qualitative blinded on/off assessment without quantitative strength testing, the three adults were evaluated primarily by open-label self-report, and detailed records were unavailable for the grandfather. Second, the paternal aunt, who is central to the inference that the response tracks with *MYBPC1* rather than *CHRND*, is also particularly susceptible to bias: she had a prior diagnosis of fibromyalgia and chronic pain, both associated with subjective outcomes and high placebo-response rates, received a relatively high dose of pyridostigmine (approximately 600 mg/day), and was assessed only by open-label self-report. Third, dosing was heterogeneous and incompletely documented across family members, ranging from 8 mg/kg/day in the proband to approximately 600 mg/day in the aunt, with the grandfather’s regimen unavailable, limiting comparisons of treatment response. Fourth, the improvement in gastrointestinal symptoms is most plausibly attributable to the generalized prokinetic cholinergic effects of pyridostigmine rather than to a disease-specific effect on the myopathy. Fifth, no electrophysiologic studies of neuromuscular transmission, such as repetitive nerve stimulation or single-fiber electromyography, were performed, so the proposed mechanism remains untested; a response to pyridostigmine alone cannot establish a secondary transmission defect or confirm a myogenic basis for the tremor. Sixth, CMYO16 may show gradual spontaneous improvement over time, which could confound assessment of longer-term benefit, although it would not explain the rapid within-dose changes and predictable wearing-off observed in the proband. Finally, p. (Arg260His) is a novel variant and has not yet been functionally characterized, although its location within the M-motif adjacent to established pathogenic residues supports pathogenicity. Taken together, these limitations indicate that our findings are preliminary and hypothesis-generating. Ideally, these preliminary observations should be followed by blinded n-of-1 or small prospective cohort studies, as such designs are feasible in ultra-rare disorders, allow patients to serve as their own controls, and help distinguish true drug effect from placebo response and observer bias.

## Conclusion

To our knowledge, this is the first report of a possible pharmacologic benefit in *MYBPC1*-associated congenital myopathy with tremor (CMYO16). In four affected members of a single family across three generations, pyridostigmine was associated with apparent, open-label improvement in muscle tone, strength, fatigability, tremor, and, concurrent improvement in gastrointestinal symptoms most likely reflects a prokinetic cholinergic effect rather than a disease-specific benefit. These findings support the hypothesis that CMYO16 may belong to the expanding group of congenital myopathies with a secondary, potentially treatable defect of neuromuscular transmission, although electrophysiologic and functional studies are needed to test this mechanism. Pending such confirmation, a carefully monitored, individualized trial of pyridostigmine may be considered in symptomatic individuals with *MYBPC1*-related myopathy, for whom no disease-directed therapy is currently available.

## Data Availability

The original contributions presented in the study are publicly available. The CHRND c.928G>A p.(Gly310Ser) variant can be found in the NCBI ClinVar repository with the accession number VCV001020812.9, and the MYBPC1 c.779G>A p.(Arg260His) variant can be found in the NCBI ClinVar repository with the accession number SCV007674154.1.
